# Popular surveillance of Chagas disease and volunteer Collaborators of Triatomine Information Posts operating in Minas Gerais, Brazil, located in municipalities at high risk of triatomine reinfestation

**DOI:** 10.1590/0074-02760260107

**Published:** 2026-11-13

**Authors:** Bruno Silva Amaral, Valéria Carla Faria Amaral, Janice Maria Borba de Souza, Genimar Rebouças Julião, Raquel Aparecida Ferreira

**Affiliations:** 1 Universidade Federal de Minas Gerais, Instituto de Ciências Biológicas, Departamento de Microbiologia, Laboratório de Interação Microrganismo-Hospedeiro, Belo Horizonte, MG, Brasil; 2 Fundação Oswaldo Cruz-Fiocruz, Instituto René Rachou, Grupo de Pesquisas Triatomíneos, Belo Horizonte, MG, Brasil; 3 Fundação Oswaldo Cruz-Fiocruz, Laboratório de Entomologia, Porto Velho, RO, Brasil

**Keywords:** entomological surveillance, health education, endemic disease control agent

## Abstract

**BACKGROUND:**

The Volunteer Collaborator (VC) of the Triatomine Information Post (TIP) is a person responsible for receiving insects suspected of being triatomines within the community.

**OBJECTIVES:**

To characterise the sociodemographic profile of VCs from TIPs; to assess their perception regarding popular surveillance and TIPs.

**METHODS:**

A semi-structured questionnaire was administered to 154 VCs working in TIPs located in rural areas at high risk of domiciliary reinfestation by triatomines in municipalities of Minas Gerais, Brazil belonging to areas at high risk of domiciliary reinfestation by triatomines.

**FINDINGS:**

40% of the VCs did not master the concept of popular surveillance. This knowledge is influenced by length of service — VCs with up to 5 years in the role performed better (p = 0.0172). No relationship was found with the other variables: education level, age group, and job training. Most VCs reported receiving few insects, due to low community demand for TIPs. They indicated that improving popular surveillance requires the implementation of health education actions, as well as communication and dissemination of TIPs within the community.

**MAIN CONCLUSIONS:**

This is the first study to highlight the need to reconfigure the role and performance of VCs, emphasising their relevance as integrators and disseminators of prevention and popular surveillance of Chagas disease (CD) in Brazil.

Currently, throughout the entire endemic area in Brazil, vector surveillance actions for Chagas disease (CD) are based on entomological surveillance which — according to the Ministry of Health manual *“Controle da Doença de Chagas, Diretrizes Técnicas”* ^(^ ^1^ ^)^ is an operational modality to be implemented in all municipalities regardless of the magnitude of entomological indicators.

Entomological surveillance in Brazil is sustained by two main pillars: active surveillance and popular surveillance (passive surveillance). Active surveillance consists of the investigation and elimination of triatomines in domiciliary units (DUs) in localities, carried out in a programmed manner by municipal/state teams.^(^ ^2^ ^,^ ^3^ ^)^ In turn, popular surveillance consists of household monitoring by residents and the notification of the finding and forwarding of insects suspected of being disease vectors^(^ ^2^ ^,^ ^3^ ^)^ to Triatomine Information Post (TIP).^(^ ^2^ ^,^ ^4^ ^)^

TIPs were conceived based on the possibility of reinfestation of localities where triatomines had been eliminated, and, consequently, on the need to monitor these localities.^(^ ^5^ ^)^ Currently, these posts are installed by health surveillance services in urban and rural areas of municipalities, mainly following geographic, population, and epidemiological criteria.^(^ ^4^ ^)^

At the time of TIP implementation in Brazil by the Superintendence of Public Health Campaigns (SPHC), the role of the TIP was defined — a member of the community, a leadership figure, preferably representing an institution (Health Unit, School etc.), who would be at the forefront of TIP activities, being responsible for receiving from the population insects suspected of being triatomines and reporting this submission.^(^ ^6^ ^,^ ^7^ ^)^

The operational steps and workflow of TIPs are described in Fig. 1. It is a circular figure, showing the need for the resident to be informed about the result of the insect examination, even when it is not a triatomine.^(^ ^8^ ^,^ ^9^ ^,^ ^10^ ^)^ This continuity of service ensures that the population remains engaged and perceives value in using TIPs. Furthermore, the community — alongside local health and education systems and public health institutions—constitutes one of the main actors in entomological surveillance of CD in Brazil.^(^ ^11^ ^)^

**Fig. 1:**
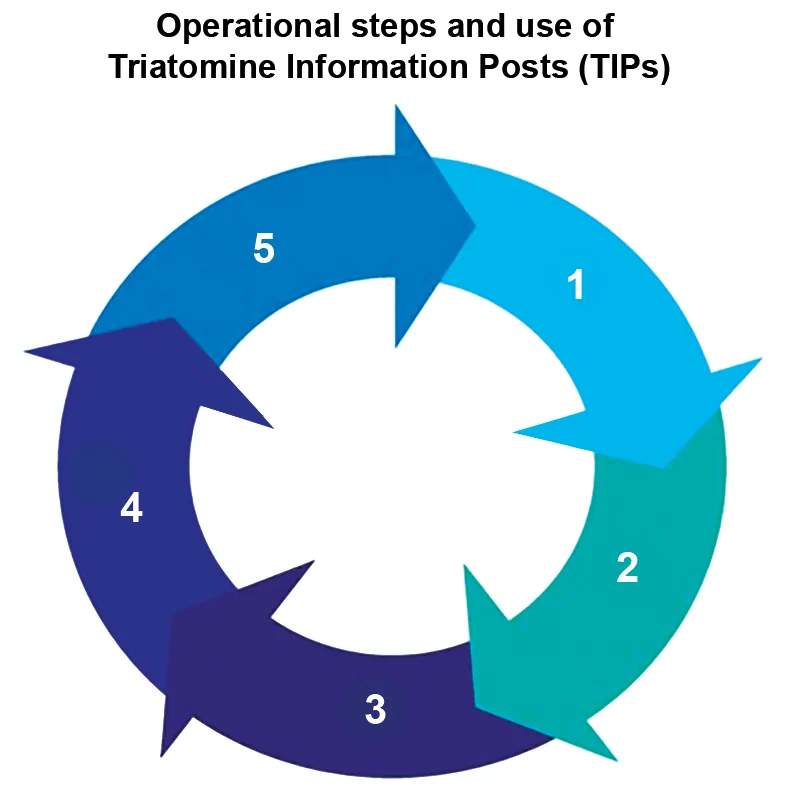
diagram presenting the stages and workflow of Triatomine Information Post (TIP) operation. In step 1, the TIP Volunteer Collaborator (VC) receives the notification and records the entry of an insect suspected of being a kissing bug delivered by the resident. In step 2, the VC communicates the receipt of the insect to the endemic disease control agent (EDCA) working in the area. In step 3, the EDCA visits the TIP and performs the identification of the insect. If it is a triatomine, in step 4 the EDCA forwards the insect to the laboratory (municipal or state) responsible for identifying and examining the insect to detect infection by *Trypanosoma cruzi*. The EDCA receives the results of the insect examination and, in step 5, visits the resident, informs them of the result, and conducts active case finding if it is a triatomine. The EDCA always visits the resident and carries out health education actions (whether the reported insect is a triatomine or not).

There is no requirement for professional training or any specific qualification to act as a Volunteer Collaborator (VC); however, acceptance and interest in joining the surveillance activities are important. The VC must receive training related to basic aspects of CD, triatomines, and entomological surveillance, as well as guidance on how to proceed with notifications of received insects.^(^ ^7^ ^)^

As stated by,^(^ ^6^ ^)^ VCs are central actors in CD surveillance; and the alignment between the work and training of these actors are indispensable aspects for the structuring and functioning of TIPs within the community. Despite this relevance, the scientific literature has never dedicated itself to carrying out any type of approach or study giving voice to these actors.

A recent study conducted in an area at high risk of domiciliary reinfestation by triatomines in Minas Gerais, Brazil, pointed to a scenario of heterogeneous distribution in the implementation and functioning of TIPs, combined with low adherence to and use of these posts by the population.^(^ ^8^ ^)^ Following this perspective, the present study — carried out in the same area as the study mentioned above — aimed to characterise the sociodemographic and operational profile of VCs; to assess VCs’ perceptions regarding popular surveillance, the use of TIPs by the community, and the performance of the endemic disease control agents (EDCAs); and finally, to assess the knowledge of these actors about aspects of CD and its vector.

## MATERIALS AND METHODS

*Ethics committee statement* - The study was submitted to and approved by the Research Ethics Committee (REC) of the Instituto René Rachou under the Certificate of Presentation for Ethical Consideration number: 37132220.8.0000.5091. Prior to data collection, five meetings were held with professionals and stakeholders from the health management within the study area. These meetings were conducted in February 2021 through an online platform and aimed to inform, raise awareness, and invite stakeholders to join the proposal.

*Study area* - According to a survey conducted by the State Health Secretariat of Minas Gerais in 2006, 123 municipalities in Minas Gerais are considered at high risk of reinfestation by triatomines in domiciliary units. The municipalities in this area belong to five health regions of the state. These are: four Regional Health Management Units (RHU): RHU of Unaí (12 municipalities), Pirapora (seven municipalities), Pedra Azul (25 municipalities), and Januária (25 municipalities); and one Regional Health Superintendence (RHS) of Montes Claros (54 municipalities), all here referred to as health regions^(^ ^8^ ^)^ (Fig. 2).

**Fig. 2:**
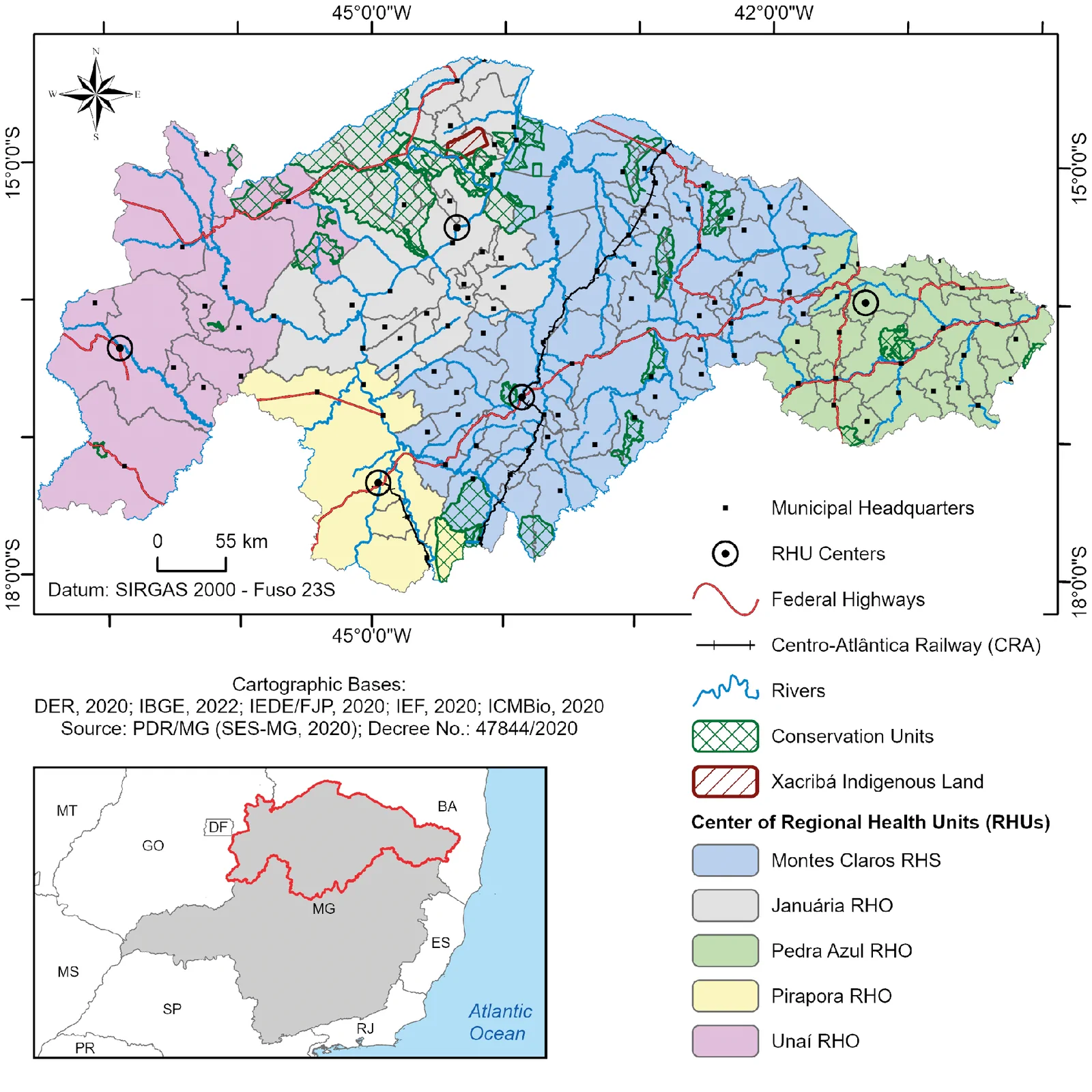
study area. Below and to the right is the map of Minas Gerais, highlighting the study area in red outline, corresponding to the 123 municipalities belonging to the four Regional Health Offices and one Regional Health Superintendence. Source: Amaral et al.^(^ ^8^ ^)^

Amaral et al.,^(^ ^8^ ^)^ working in this high-risk area, in the same time and space as the present study, conducted an extensive survey of TIPs implemented in the area. From this survey, it was shown that in the high-risk area, 339 TIPs had been installed, 222 in rural areas of 50 municipalities and 117 in urban areas. In the present study, it was decided to focus on volunteer collaborators of TIPs in rural areas. This focus is due to the fact that these localities present high social vulnerability, with inhabitants living in unhealthy environments and presenting precarious habits and living conditions,^(^ ^12^ ^)^ thus being exposed to a greater probability of contact with triatomines and remaining the segment of the Brazilian population most susceptible to Chagas infection.^(^ ^13^ ^,^ ^14^ ^)^

*Data collection* - The present study was conducted during the Coronavirus disease 19 (COVID-19) pandemic. For this reason, it was not feasible for the project team to administer the questionnaires directly to the VCs. Therefore, the team invited EDCAs, via email, from the 50 municipalities in the area that had active TIPs in rural areas, and conducted an online training to qualify these professionals to administer the questionnaire. Five training meetings were held, one per health region. All meetings took place in November 2021 and lasted approximately two hours each. On this occasion, participants were presented with a summary of the proposal, the Informed Consent Form (ICF), and the questionnaire. The professionals were instructed to read the ICF to the research participants, collect signatures in two copies (one to be returned to the researchers), and to read and complete the questionnaire. They were instructed not to interfere with any responses and only to clarify participants’ doubts after completion of the instrument. It is noteworthy that this method of administration was reviewed, adjusted, and approved by the REC of the Instituto René Rachou.

After training the EDCAs to administer the questionnaire to TIP VCs, the recording of the meeting was sent to them for consultation. In addition, the project team remained available to clarify any doubts. The questionnaire was administered between November 2021 and March 2022. After completion, the professionals sent the questionnaires and signed ICFs by mail to the research team.

*Data collection instrument and analysis* - Between August and September 2021, 30 pilot questionnaires were administered in person to VCs in the municipality of Cláudio, located in the Midwest region of Minas Gerais, Brazil. This municipality was selected based on convenience and previously established cooperation. At this stage, in-person administration was chosen due to ease of access to professionals in the area and the understanding that this approach would be more effective for testing purposes. Based on this, the suggested improvements were incorporated into the final semi-structured questionnaire, which was subsequently applied in the study area.

The semi-structured questionnaires used in the study comprised three central axes of questions: sociodemographic profile of participants; knowledge related to CD and its vectors; knowledge/perception about popular surveillance; and knowledge/perception about the role of the EDCA. The questionnaire contained 24 questions, including closed, open, and semi-open questions.

All received questionnaires had their responses tabulated and organised in Microsoft Excel^®^. Responses to closed questions were quantified, and tables and graphs were created using GraphPad Prism version 8.02.

For the analysis of open-ended questions, three researchers read and analysed the responses and grouped them into categories, considering similarities in meaning. Responses for which it was not possible to assign meaning or that were too general and nonspecific were disregarded. For the question related to participants’ professions, responses were grouped according to their frequency as follows: rural workers — including those who reported being farmers or performing other rural-related occupations; nurses — including both technical- and higher-level nursing professionals; individuals who reported working as Community Health Agents (CHAs) and EDCAs; and others — a category that included less frequent professions such as domestic worker, homemaker, driver, among others.

*Statistical analysis* - Several analyses of dependence between variables were performed. The relationships evaluated were those for which there was an explanatory hypothesis regarding the possible influence of one variable on another. Thus, the following were assessed: the influence of length of service on knowledge of the concept of passive surveillance; the influence of training on knowledge of the concept of passive surveillance; the relationship between age group and length of service; age group and knowledge of popular surveillance; age group and participation in training; education level and mastery of the concept of popular surveillance; and training and education level. The number of insects received was also evaluated according to municipalities from different health regions. For all these analyses, the chi-square test of independence was used. Finally, it was evaluated whether the type of installation site (*i.e.*, whether it was a residence, health unit etc.) of TIPs influenced the number of insects received; for this analysis, Fisher’s test was used. A 95% significance level was adopted for all tests.

## RESULTS

*Sociodemographic profile* - At the time of data collection, there were 154 volunteer collaborators working in 154 TIPs across 19 municipalities. Compared to the previous survey by Amaral et al.,^(^ ^8^ ^)^ 68 TIPs in 31 municipalities had no VCs. Of the 154 TIP VCs, 80 responded to the questionnaire, corresponding to 51.9%. Information on the number and percentage of respondents according to the health region and number of associated municipalities is described in Table I. The sociodemographic profile of TIP VCs can be observed in Table II.

**TABLE I:** Number and percentage of respondents according to the number of Volunteer Collaborators (VCs) by Regional Health Management Unit/Superintendence and municipalities

Health region	Number of municipalities	Number of VCs	Number of respondents	Percentage (%)
Januária	08	29	29	100.0
Montes Claros	05	90	25	27.8
Pedra Azul	01	02	01	50.0
Pirapora	03	20	20	100.0
Unaí	02	13	05	38.5
Total	19	154	80	51.9

**TABLE II:** Sociodemographic profile of Triatomine Information Post (TIP) Volunteer Collaborators (VCs) participating in the study (absolute values and percentages)

Variable		Absolute (N)	Percentage (%)
Sex	Male	28	35.00
	Female	52	65.00
Age group (years)	18-29	5	6.25
	30-39	25	31.25
	40-49	21	26.25
	50-59	15	18.75
	> 60	14	17.50
Education level	Iliterata	3	3.80
	Incomplete primary education	14	17.72
	Complete primary education	11	13.92
	Incomplete secondary education	2	2.53
	Complete secondary education	33	41.77
	Incomplete higher education	1	1.27
	Complete higher education	14	17.72
	Postgraduate education	1	1.27
Occupation	Rural workers	23	28.75
	Nurses	22	27.50
	Community Health workers	10	12.50
	Endemic Disease Control Agents	7	8.75
	Others	18	22.50
Lenght of service	< 1 year	28	35.00
	1-2 years	5	6.25
	3-5 years	9	11.25
	6-10 years	8	10.00
	> 10 years	29	36.25
	Others	1	1.25
	Missing	1	1.25
Training for the role	Yes	39	48.75
	No	39	48.75
	Missing	1	1.25

N: number.

An association was observed between the variables age group and length of service of the participants (p = 0.0075), as presented in Table III. For each length-of-service category, there are two internal subcolumns: the first corresponds to the observed responses and the second to the expected values by chance for each category (according to the chi-square test of independence). There are more VCs with less than one year of service in the 30-39 age group than would be expected by chance. On the other hand, there are fewer VCs in the 50 years or older age group. Additionally, there are fewer VCs with more than 10 years of service in the 30-39 age group and more than expected by chance in the group over 50 years of age. In contrast, no association was observed between participants’ age group and participation in training (p = 0.6990), nor between having undergone training and level of education (p = 0.3857).

**TABLE III:** Length of service in Triatomine Information Post (TIP) according to the age group of Volunteer Collaborators (VCs)

Age group (years)	> 1 year		1-2 years		3-5 years		6-10 years		< 10 years		Total
18-29	0	1.5	0	0.2	0	0.4	01	0.4	3	1.5	4
30-39	15	9.1	04	1.6	01	2.6	02	2.6	03	9.1	25
40-49	07	7.6	0	1.4	04	2.2	02	2.2	08	7.6	21
50-59	02	5.1	01	0.9	02	1.5	02	1.5	07	5.0	14
< 60	01	4.7	0	0.8	01	1.4	01	1.4	10	4.7	13
Total	28		05		08		08		28		77

*Aspects related to CD and its vectors* - Supplementary data (Table) presents the data related to the participants’ responses regarding several aspects of CD and its vectors. The participants demonstrated a high level of knowledge about the biological and behavioural aspects of triatomines [Supplementary data (Table)]. However, regarding the mode of parasite transmission, only 10% of the participants recognised that *T. cruzi* can also be transmitted through triatomine urine, and 16.25% stated that parasite transmission occurs through the insect bite.

*Knowledge and perception about popular surveillance, community involvement, and TIPs* - With the exception of one participant, all others (79) considered the TIP in which they worked to be important. Regarding the concept of popular surveillance, the questionnaire presented four possible answers, and the participants’ understanding of this concept is described in Table IV.

**TABLE IV:** Participants’ responses regarding the concept of popular surveillance

Response/Alternative	Number of respondents (N)	Percentage (%)
Community participation in insecticide spraying actions in households (intra- and peridomicile)	4	5.00
Community participation in reporting and submitting suspected triatomine insects to TIPs	48	60.00
Household visits by endemic disease control agents (EDCA)s) or Chagas disease (CD) agents	9	11.25
Educational and informational actions for the population carried out by EDCAs	17	21.25
Invalid response	02	2.50
Total	80	100.00

There was a difference in the mastery of the concept of popular surveillance between professionals with up to five years of service and those with more than five years, with the former showing a higher rate of correct responses (Table V). On the other hand, training in the role did not influence the mastery of the evaluated knowledge (Table V). Likewise, there was no association between age group or level of education and knowledge about popular surveillance (p = 0.6577 and p = 0.2728, respectively).

**TABLE V:** Comparison of the influence of length of service and training in the role on the mastery of the concept of popular surveillance

Comparison	Correct answers (N)	Incorrect answers (N)	p-value
≤ 5 years of service × concept of popular surveillance	30	10	0.0172
> 5 years of service × concept of popular surveillance	16	18	
Trained collaborator	27	09	0.0505
Untrained collaborator	18	17	

N: number.

Regarding the receipt of insects per year at TIPs, 34 participants (42.5%) reported receiving many insects, whereas 46 (57.5%) reported receiving a small number of insects per year. When asked about the reasons for not receiving many insects at TIPs, 36 participants (78.3%) mentioned some difficulties; among these, 18 cited lack of information and knowledge among the population as the most likely cause. Of these, four specifically mentioned lack of knowledge/information and the population’s inability to recognise the vector, and one participant mentioned lack of knowledge about what TIPs are and their role. The remaining participants were general in mentioning lack of knowledge, making it impossible to infer what type of knowledge they were referring to.

Nine of the 36 participants attributed the low number of insects received to the population not recognising the importance of TIPs and CD as a public health issue. Five participants highlighted as a cause the low rate of triatomine infestation in the localities and the infrequent encounters of insects by residents. Two participants pointed out the lack of dissemination about TIPs in the communities. One participant highlighted that TIPs are located in distant areas, and another mentioned as a problem the centralisation of TIPs in the zoonosis sector.

It was evaluated whether the type of establishment (or location) hosting the TIPs influenced the number of insects received and reported (Table VI). For this, information on the location of TIP installation in the territories — primary health care units (PHCUs) and residences — was cross-referenced with participants’ responses regarding whether they received many or few insects. According to the data, apparently, there is no such relationship.

**TABLE VI:** Comparison between the type of location hosting Triatomine Information Post (TIP) and the receipt of insects

TIP installation site	High number of insects received (N)	Low number of insects received (N)	p-value
Primary Health Care Unit	21	21	0.2175
Household	09	19	

N: number.

Regarding the receipt of insects at TIPs according to the RHU, when the statistical test was applied, it was observed that in municipalities from the Januária and Unaí health regions, more insects were received than expected by chance. On the other hand, in municipalities from the Pirapora and Montes Claros health regions, fewer insects were received than would be expected by chance (Table VII). According to the chi-square test of independence, the variables are dependent (p = 0.0072).

**TABLE VII:** Number of participants from the health regions who reported receiving many insects at Triatomine Information Post (TIP) (Yes) and few insects (No), and the expected values by chance for these two responses

Health region	Yes	Yes (Expected)	No	No (Expected)
Januária	16	12.5	13	16.5
Pirapora	06	8.6	14	11.4
Unaí	05	2.2	0	2.8
Montes Claros	07	10.8	18	14.2

Figure 3 presents the frequency of the main causes that could lead a TIP to become unproductive, according to the perspective and perception of the participants. In the questionnaire, participants could provide more than one cause, and these were grouped by similarity of meaning into five categories. Communication/dissemination was the category that grouped the largest volume of responses 36 records. Responses that pointed to lack of information, communication, guidance, and dissemination of TIPs as a problem and cause of TIP inactivity were included in this category. The second category with the highest number of responses was lack of popular participation (N = 27). This category included statements mentioning lack of interest, lack of community participation, and population complacency regarding the use of TIPs. Five participants mentioned that the cause of TIP inactivity would be the absence or reduction of triatomines in the locality (Fig. 3 — Absence/Reduction in the Occurrence of Triatomines). Three participants mentioned that the population preferred to deliver insects to PHCUs or health posts due to the perception of faster feedback regarding insect identification (Fig. 3 — use of other delivery locations). Three participants reported failures in the surveillance service in issuing and delivering insect test results to the population, stating that this would lead to distrust and disengagement regarding TIP use. Ten additional responses of random content were recorded, not following a common line of reasoning, not fitting into any category, or not presenting a clear meaning that could be interpreted. Finally, seven participants did not answer this question.

**Fig. 3:**
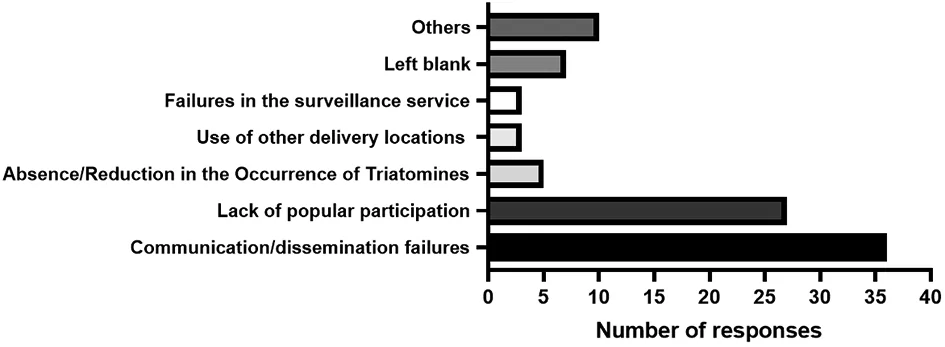
frequency of the main causes that could lead a Triatomine Information Post (TIP) to become unproductive, according to the perspective and perception of the participants.

For 61 participants (76.25%), the population made little use of TIPs, while 23.75% of participants considered the opposite. The reasons listed by participants for why the population would not participate in CD surveillance are presented in Fig. 4.

**Fig. 4:**
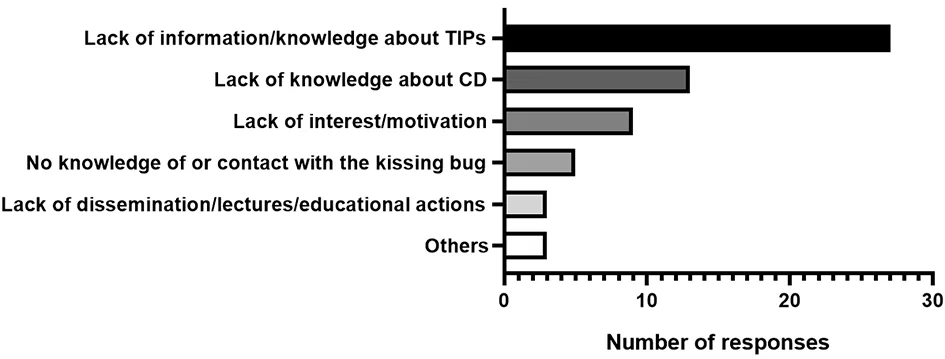
the main reasons reported by the participants for the population’s non-participation in Chagas disease (CD) surveillance.

Participants could describe more than one proposition related to strategies that could be used to increase population participation and engagement in entomological surveillance of CD. The most frequent are highlighted in Table VIII.

**TABLE VIII:** Main strategies and resources highlighted by participants as elements capable of mobilising their communities

Strategy/Resource	Number of recommendations (N)
Meetings, lectures, and other strategies involving the community and health professionals	29
Educational actions: campaigns, events, and household visits directed at residents	20
General dissemination/communication about Triatomine Information Post (TIP)	16
Guidance provided by endemic disease control agents (EDCAs) to residents	06
Training and continuing education of health professionals	04
Meetings and partnerships among services, encouraging surveillance	03
Government resources and incentives, including hiring professionals	03
Improvement of conditions and access to TIPs	02
Active search	01

Participants were asked to provide suggestions for improving popular surveillance, that is, population participation in CD control actions. In a very expressive manner, 64 participants mentioned some form of dissemination and awareness-raising among residents about the disease, the vector, and TIPs. The strategies mentioned included lectures, meetings, distribution of pamphlets, guidance during religious services and other prominent community settings, and specific actions for residents in rural areas, among others. Four participants highlighted the need for greater commitment from professionals during household visits; four emphasised the need for alignment between health surveillance and PHC, health councils, and schools; and finally, one participant mentioned that public authorities should be responsible for community engagement in CD control.

*VCs’ perspective on the role of EDCAs in popular surveillance of CD* - Regarding the importance of monthly visits by EDCAs to TIPs, 73 participants (91.25%) considered this action relevant, while seven (8.75%) disagreed. Among those who considered the visits important, 61 provided reasons for this relevance. These responses were analysed and, based on similarity of arguments, categorised into five major themes presented in Fig. 5. The category with the highest number of responses (N = 21) was health education. This category included responses in which participants argued that EDCA visits to TIPs are crucial for bringing assistance, information, and guidance about the kissing bug and TIPs to the community.

**Fig. 5:**
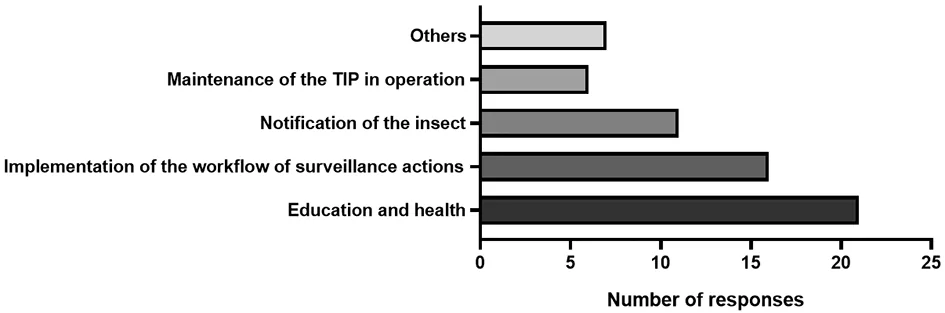
main reasons listed by the participants that justify the importance of the endemic disease control agent (EDCA) monthly visits to the Triatomine Information Post (TIP).

The second category with the highest number of responses was execution of surveillance workflows (N = 16). This category included responses highlighting that EDCA visits to TIPs fulfil surveillance workflows, meet service targets, and contribute to prevention and vector control actions. Next, with 11 responses, the insect notification category encompassed responses that directly linked the importance of monthly EDCA visits to the collection and examination of the vector. Six participants highlighted that the importance of monthly EDCA visits to TIPs is fundamental for maintaining TIP functioning and community engagement in their use. Finally, seven participants mentioned other reasons or statements that could not be assigned to a specific category, as they were too general and nonspecific.

Still regarding the actions of EDCAs, participants were asked whether they considered any of these professionals’ actions unnecessary, and only one participant (1.25%) indicated that there was, mentioning the activity “basic guidance” as unnecessary. Complementing this perspective on EDCA work, participants were asked which factors and actions could improve the activities of EDCAs and TIPs in the context of CD surveillance. There was a wide variety of responses, making precise categorisation difficult, as many answers revolved around aspects that did not always directly relate to the question asked.

Even so, some improvements were frequently mentioned by participants, the main one being the implementation of dissemination campaigns about the disease, vectors, and TIPs through the use of pamphlets, posters, and lectures, cited by 28 respondents. Another aspect clearly highlighted by 16 participants was the need to improve surveillance services [availability of vehicles and transportation, personal protective equipment (PPE) and support from management and local governments]. Eight participants emphasised or mentioned the need for training of EDCAs.

The following weaknesses were also cited: the need for more frequent EDCA visits to TIPs; the need for EDCAs to promote TIPs during household visits; expansion of the TIP network to more distant locations; development of joint actions with schools, associations, and PHC; greater speed in EDCA actions and in returning results; prioritisation of visits to more vulnerable and rural areas; and, finally, greater commitment and involvement of the population.

## DISCUSSION

In Brazil, popular health surveillance (PHS) emerged after the COVID-19 pandemic, being a contemporary concept still under construction,^(^ ^15^ ^)^ born from the dialogue between the Unified Health System (SUS), popular health education, and social movements. The purpose of popular surveillance is to value local knowledge alongside technical-scientific knowledge and to mobilise communities to become protagonists in defending health according to the characteristics of their territories.^(^ ^15^ ^)^

*Vigia, Povo! Um guia de vigilância popular em saúde* ^(^ ^15^ ^)^ presents and discusses different community experiences in Brazil related to issues involving environment and work. Here, the present study focused on exploring aspects related to community experience in vector control of CD.

In Brazil, surveillance with popular participation in CD is supported and anchored by TIPs, through which field actions are triggered and executed, with entomological surveillance currently being practically restricted to them.^(^ ^11^ ^)^ From this perspective, working in one of the regions of greatest vulnerability for CD in the world, the present study focused on understanding the profile and performance of TIP VCs, who occupy a central role in popular surveillance, being strategic actors for understanding the structuring and use of TIPs in the territories. VCs are directly connected to the other actors who sustain and operate TIPs. As intermediaries between the community and health institutions/surveillance, they hold information and perceptions about community engagement and the use of TIPs by residents. Finally, they interact with EDCAs by communicating the arrival of insects at TIPs and during the agents’ monthly visits. Below is the discussion regarding the profile and performance of VCs, their perception and role in popular surveillance, and their perceptions of different actors involved in this process.

*Sociodemographic profile and performance of VCs* - A decline in the number of VCs in the study area was observed when compared to the survey conducted by Amaral et al.^(^ ^8^ ^)^ In that study, at the time of data collection, the authors assumed that all TIPs declared as active by endemic disease coordinators would have an associated VC. However, it appears that coordinators did not consider the requirement of having a VC when stating that a TIP was active. On the other hand, it cannot be ruled out that, between data collection periods, some collaborators may have left TIPs. In fact, Amaral et al.,^(^ ^8^ ^)^ during focus groups, captured statements from coordinators indicating that VCs abandoned TIPs in several municipalities, leading to their inactivity.

Taniguchi^(^ ^16^ ^)^ provides a reflection that aligns closely with the findings of this study, associating the decline in volunteering in general with the increase in female employment. Considering that women were the majority among TIP VCs in this study, this may be a possible cause of TIP abandonment and deactivation in the study area. Furthermore, women represented 57% of all global volunteer work,^(^ ^17^ ^)^ which aligns with the finding of this study showing that 65% of TIP VCs were female.

The education level of VCs in this study also aligns with data from the PNAD survey released by IBGE in 2024,^(^ ^18^ ^)^ which showed that 56% of the Brazilian population aged 25 years or older has completed basic education (*i.e.*, completed high school), a profile expected for the participants of this study.

The professions of VCs were quite diverse, with rural workers and nurses together representing the majority of participants. These findings are consistent with the study setting — rural areas — where many residents work in agriculture and livestock.^(^ ^19^ ^)^ On the other hand, nurses and agents are professions related to care and health, and a high number of VCs in these professions is also expected, since TIPs are preferably installed in health units.^(^ ^6^ ^)^

The predominant age group of VCs in this study was between 30 and 49 years, which was expected, as these are adults in an active stage of life. Regarding length of service as VCs, there was a polarisation of data at two extremes — less than one year and more than 10 years — together accounting for more than 70%. Additionally, an association was observed between the 30-39 age group and VCs with less than one year of service, and between those over 50 years of age and VCs with more than 10 years of service. This latter association suggests that some volunteers joined popular surveillance at a younger age and continue to collaborate. Future studies focusing on this specific profile may further explore the characteristics and motivations behind sustained engagement, pointing to strategies that could be replicated in popular surveillance and in the selection of future VCs.

Regarding training, it is noteworthy that nearly 49% of VCs had never received training for this role. Amaral et al.,^(^ ^8^ ^)^ interviewing endemic disease coordinators from the same municipalities, also showed through their statements that VCs are not adequately trained to act in surveillance with popular participation, highlighting the need for investment in training and qualification of these actors.

*Aspects related to CD and its vectors* - As previously mentioned, there are no studies in the scientific literature conducted with VCs from TIPs, making it impossible to establish appropriate comparisons regarding the knowledge of CD and its vectors among these actors and participants with similar roles in community-based CD surveillance addressed in other studies. However, the scientific literature includes several studies investigating and discussing the knowledge and attitudes of schoolchildren regarding CD and its vectors,^(^ ^20^ ^,^ ^21^ ^,^ ^22^ ^)^ as well as studies on the knowledge, perceptions, attitudes, and practices of populations from endemic areas in different countries concerning CD, vector control, access to healthcare, healthcare practices, among other aspects.^(^ ^14^ ^,^ ^23^ ^,^ ^24^ ^,^ ^25^ ^)^ Finally, there are also studies evaluating these aspects among healthcare professionals.^(^ ^26^ ^,^ ^27^ ^,^ ^28^ ^,^ ^29^ ^)^

In the present study, the VCs demonstrated a high level of knowledge regarding aspects of triatomine biology and physiology, which is an important finding considering that nearly half of them had never participated in any type of training. However, regarding the mode of parasite transmission, only a few recognised insect urine as a route of contamination, while some stated that transmission occurs through the insect bite itself. This lack of knowledge and confusion is frequently observed among other social actors as well,^(^ ^20^ ^,^ ^21^ ^,^ ^22^ ^,^ ^24^ ^)^ highlighting the need for this topic to be more thoroughly addressed in training courses, lectures, science communication initiatives, and social media campaigns.

*Knowledge and perception about popular surveillance* - As previously described, popular surveillance of CD is defined as the modality of entomological surveillance carried out by the general population, based on the collection of insects suspected of being triatomines and their subsequent submission to TIPs.^(^ ^5^ ^)^ In this study, 60% of participants demonstrated effective knowledge of this concept, while the others showed conceptual confusion. No association was found between this knowledge and education level, age group, or having received training.

On the other hand, knowledge of the concept was influenced by length of service, with VCs who had up to five years of service demonstrating greater accuracy than those with longer service. As no relationship was found with other variables, a possible explanation for this finding may be a decline in motivation over time. Indeed, a theory in the literature on job design suggests that, as tenure increases, employees may become more bored and less motivated, which can offset gains in experience in terms of overall performance.^(^ ^30^ ^)^ However, further studies are needed to better clarify this issue.

Abad-Franch et al.,^(^ ^31^ ^)^ through a systematic review, reinforce the effectiveness of popular surveillance in controlling CD, pointing to it as more effective and less costly than methods such as active search and insecticide spraying. In contrast, in the present study, most VCs reported receiving few insects, as the population makes little use of TIPs. Furthermore, no relationship was found between the type of establishment hosting TIPs and the number of insects received, indicating that this is not a determining factor.

In this context, the most frequently reported explanation for the low number of insects received and low use of TIPs was the lack of information and knowledge among the population about triatomines and TIPs themselves. This scenario suggests a weakness in CD control in the study region, since the population is the central and indispensable element for the success of popular surveillance. In addition to low engagement, the data point to a probable gap in knowledge among this group. These findings align with those of Amaral et al.,^(^ ^8^ ^)^ who reported that, from the perspective of endemic disease coordinators, one of the main challenges to TIP use and sustainability is the lack of community engagement and awareness.

When comparing insect receipt at TIPs across municipalities from different health regions, it was observed that some regions received more insects than expected by chance (Unaí and Januária), while others received fewer (Montes Claros and Pirapora). Amaral et al.^(^ ^8^ ^)^ showed that a higher percentage of TIPs are active in rural areas of the latter regions compared to the former. This highlights an important point — it is not enough to have TIPs in place; the population must be mobilised and engaged in using them. Indeed, participants in this study emphasised that the population generally makes little use of TIPs.

Dias and Dias^(^ ^32^ ^)^ argue that popular surveillance of CD requires continuous and specific attention, especially in areas where peridomestic or sylvatic triatomines periodically invade and colonise houses — such as in the municipalities included in this study, classified as high-risk areas for domiciliary reinfestation. The authors also note that flexible and context-adapted strategies can be effective when there is harmonious cooperation between communities and institutions.

Low demand for TIPs leads to low productivity of these posts. In this regard, VCs identified lack of communication and dissemination, followed by lack of popular participation, as the main reasons for TIP inactivity. These same factors were also identified by endemic disease coordinators in Amaral et al.^(^ ^8^ ^)^ Together, these findings point to a concerning scenario: if TIP inactivity is linked to lack of communication, dissemination, and community engagement — and low population adherence is already observed — there is a risk of worsening CD surveillance, potentially leading to an increase in cases.

As strategies to increase community participation and engagement, VCs suggested various health education actions, especially campaigns to disseminate information about CD, TIPs, and prevention. These measures are also highlighted by Moreno et al.^(^ ^6^ ^)^ as fundamental for strengthening participatory surveillance. It is important to develop actions that empower the population to feel like protagonists, as a key principle of popular surveillance is enabling individuals to become active agents in monitoring and identifying health issues.^(^ ^15^ ^)^

An important milestone in Brazil was the creation of the National Policy on Popular Health Education.^(^ ^33^ ^)^ Based largely on Paulo Freire’s principles of popular education, it proposes that health care production be carried out with people and communities, not just for them — promoting participatory and transformative health practices that recognise the population as active subjects in care. In this context, teachers and educators are essential community actors who should be involved in endemic disease surveillance processes within their territories. The continuing education of these actors is another aspect that deserves special attention. Along these lines, a study conducted in the municipality of Teresina, Piauí, showed that continuing education for teachers on aspects related to CD and its vectors significantly increased the acquisition of knowledge among these actors, thereby strengthening awareness of the topic and consequently enhancing their role in the surveillance and monitoring of triatomines.^(^ ^34^ ^)^

*VCs’ perspective on the role of EDCAs in popular surveillance of CD* - As highlighted, EDCAs are part of municipal/state health teams and, in the context of CD control, act directly in active surveillance but also participate in popular surveillance.^(^ ^7^ ^,^ ^26^ ^)^ In active surveillance, they conduct programmed household visits, active searches for triatomines, and insecticide spraying when necessary.^(^ ^3^ ^,^ ^4^ ^,^ ^35^ ^)^ They also act as health educators, guiding and mobilising the population regarding CD, vectors, prevention, and the use of TIPs.

In popular surveillance, EDCAs carry out monthly visits to TIPs or respond when contacted by VCs for the collection of insects delivered by residents.^(^ ^5^ ^,^ ^26^ ^)^ In this study, most VCs considered monthly EDCA visits to TIPs essential, particularly for education and guidance actions directed at the population. However, it is important to note that health education actions are not limited to TIP visits and are also conducted during routine household visits.

VCs also highlighted that EDCA visits are important for maintaining surveillance workflows and achieving service goals. Several responses also linked the importance of these visits to insect notification and collection, demonstrating that VCs understand EDCA responsibilities and value their role in CD control and surveillance.

As strategies to improve EDCA performance, participants emphasised the need to intensify dissemination and education campaigns about CD, TIPs, and prevention. This perception is relevant, as EDCAs are key actors in directly interacting with the population and should lead educational actions that promote autonomy and collective health awareness.^(^ ^36^ ^)^

VCs also highlighted the need to improve surveillance services (availability of vehicles, transportation, PPE, and management support) and to provide better training for EDCAs. Service precariousness has already been widely discussed in previous studies,^(^ ^26^ ^,^ ^37^ ^)^ as has the need for EDCA training on CD and vectors.^(^ ^26^ ^)^ Finally, as emphasised by Amaral et al.,^(^ ^8^ ^)^ for a service to function effectively, the actors involved must be properly trained and updated in their roles.

The National Health Surveillance Policy^(^ ^38^ ^)^ highlights the need for greater participation of social movements and communities in surveillance. However, it is important to emphasise that popular health surveillance is not intended to replace the role of the State, but rather to complement and integrate it. In this context, an important reflection concerns the volunteer work carried out by community collaborators of the TIPs.

The way entomological surveillance of CD was conceived and structured in Brazil, including popular surveillance,^(^ ^5^ ^)^ dates back to the period when the country was transitioning out of the military regime. The 1980s were marked by economic crisis, high inflation, low growth, increased poverty, and fiscal instability.^(^ ^39^ ^)^ From that decade onward, Brazil underwent profound transformations in its social, economic, and structural context.^(^ ^40^ ^)^ It is worth highlighting the promulgation of the Federal Constitution, which represented a major social milestone by expanding social and labour rights and ensuring universal access to health, education, and social security.^(^ ^41^ ^)^

Moving into the 2000s, Brazil experienced economic growth associated with the expansion of redistributive social policies, resulting in reduced poverty, decreased income inequality, increased consumption among lower-income populations, and expansion of higher education, ultimately leading to greater social inclusion.^(^ ^40^ ^)^ This inclusion has resulted in individuals spending most of their time engaged in paid work, which establishes a limit on the time available for other activities. Thus, employment status may significantly influence both the likelihood and the extent of volunteering.^(^ ^42^ ^)^ The number of working hours is therefore commonly considered a limiting factor for volunteer work.^(^ ^42^ ^)^ Consequently, it is expected that in the coming years the availability of TIP community collaborators will worsen, making it increasingly difficult to recruit individuals to perform this crucial role in the popular surveillance of CD in Brazil.

Furthermore, the use of new technologies, such as the internet and social media, may impact the role of the community collaborator, as many individuals have begun using insect identification platforms, sending photos directly to health services or taxonomic experts instead of using TIPs.^(^ ^43^ ^,^ ^44^ ^)^ These platforms have contributed to popular surveillance of CD, while also intensifying the dispersion of information and leading to fragmentation in data integration regarding population engagement with official identification services — an issue that deserves attention from higher levels of health and surveillance management. Additionally, when dealing with triatomines, it is necessary for the insect to reach the health service so that parasitological examination can be performed.^(^ ^8^ ^,^ ^43^ ^)^

Through this pioneering study, we conclude that one of the major challenges that popular surveillance in Brazil will need to address is the reconfiguration of the role and figure of the TIP community collaborator. These changes should emphasise the relevance of this actor as an integrative and disseminating agent in the popularisation of science and in the prevention and control of CD, as well as the development of strategies to enhance community engagement and awareness.

## SUPPLEMENTARY MATERIALS

Supplementary material

## Data Availability

The data generated in the present study are not publicly available but may be obtained upon request from the corresponding author.

## References

[1] MS/FNS - Ministério da Saúde/Fundação Nacional de Saúde (1994). Controle da doença de Chagas: diretrizes técnicas.

[2] Dias JVL, Queiroz DMM, Diotaiuti L, Pires HHR (2016). Conhecimentos sobre triatomíneos e sobre a doença de Chagas em localidades com diferentes níveis de infestação vetorial. Cien Saude Colet.

[3] MS/SVS - Ministério da Saúde/Secretaria de Vigilância em Saúde (2012). Nota Técnica nº 36/2012. Orientações sobre vigilância entomológica e a utilização de inseticida de ação residual no controle de triatomíneos: vetores da doença de Chagas.

[4] MS/SVS - Ministério da Saúde/Secretaria de Vigilância em Saúde. Coordenação-Geral de Desenvolvimento da Epidemiologia em Serviços (2019). Guia de Vigilância em Saúde: Vol. único.

[5] MS/SCSP - Ministério da Saúde/Superintendência de Campanhas de Saúde Pública (1980). Manual de normas técnicas da campanha de controle da doença de Chagas.

[6] Moreno EC, Baracho L (2000). Vigilância epidemiológica no Programa de Controle da Doença de Chagas em Minas Gerais, Brasil (1984-1998). Cad Saude Publica.

[7] Souza JMB, Diotaiuti LG, Dias JCP (2019). Vigilância da doença de Chagas: manual técnico das atividades de controle de triatomíneos.

[8] Amaral VCF, Freitas MVS, Paulo GL, Barbosa SE, Souza JMB, Amaral BS (2025). Community-based surveillance of Chagas disease: characterization and use of Triatomine Information Posts (TIPs) in a high-risk area for triatomine reinfestation in Latin America. PLoS Negl Trop Dis.

[9] Villela MM, Souza JB, Mello VP, Azeredo BVM, Dias JCP (2005). Vigilância entomológica da doença de Chagas na região centro-oeste de Minas Gerais, Brasil, entre os anos de 2000 e 2003. Cad Saude Publica.

[10] Silveira AC, Yadón ZE, Gürtler RE, Tobar F, Medici AC (2006). Descentralización y gestión del control de las enfermedades transmisibles en América Latina.

[11] Dias JCP (2000). Vigilância epidemiológica em doença de Chagas. Cad Saude Publica.

[12] Vinhaes MC, Dias JCP (2000). Doença de Chagas no Brasil. Cad Saude Publica.

[13] Miranda DLP, Reis MG, Ribeiro GJS, Lanza FC, Reis RB, Silva MMSN (2018). Soroprevalência da doença de Chagas em doadores de sangue de serviço de referência no estado da Bahia. In: 54th Congress of the Brazilian Society of Tropical Medicine. Sociedade Brasileira de Medicina Tropical.

[14] Souza ICA, Rodrigues FCS, Araújo AP, Souza JMB, Diotaiuti LG, Ferreira RA (2023). Moradores de áreas rurais de municípios mineiros endêmicos para a doença de Chagas: ideias e concepções sobre a doença, os vetores e os serviços de saúde. Cad Saude Colet.

[15] FIOCRUZ - Fundação Oswaldo Cruz (2023). Vigia, povo! Guia de vigilância popular em saúde: protagonismo popular na defesa da vida.

[16] Taniguchi H (2006). Voluntariado masculino e feminino: diferenças de gênero nos efeitos das características de emprego e família. Nonprofit Volunt Sect Q.

[17] Borromeo K (2021). Beyond averages: do gender disparities exist in volunteering?. https://knowledge.unv.org/evidence-library/promoting-gender-equality-through-volunteering-in-national-or-subnational-policies-and-frameworks.

[18] IBGE - Instituto Brasileiro de Geografia e Estatística (2025). PNAD Contínua: educação 2024.

[19] Buainain AM, Dedecca CS (2008). Emprego e trabalho na agricultura brasileira.

[20] Cabrera R, Mayo C, Suárez N, Infante C, Náquira C, García-Zapata MT (2003). Conocimientos, actitudes y prácticas sobre la enfermedad de Chagas en población escolar de una zona endémica del Perú. Cad Saude Publica.

[21] Oliveira NWC, Salles HCR, Vicq FM, Ferreira RA, Mambrini JVM, Diotaiuti L (2023). Conhecimentos sobre a doença de Chagas entre escolares de dois municípios de Minas Gerais, Brasil. Cad Saude Colet.

[22] Vivas ALP, Ferreira RA, Oliveira ER, Ianes IM, Barbosa SE (2022). Avaliação do conhecimento sobre a doença de Chagas em escolares das zonas rural e urbana de municípios endêmicos em Minas Gerais. Physis (Rio J).

[23] Parisi S, Navarro M, Du Plessis JD, Shock JP, Michel BA, Espinoza ML (2020). “We have already heard that the treatment doesn’t do anything, so why should we take it?”: a mixed method perspective on Chagas disease knowledge, attitudes, prevention, and treatment behaviour in the Bolivian Chaco. PLoS Negl Trop Dis.

[24] Salm A, Gertsch J (2019). Cultural perception of triatomine bugs and Chagas disease in Bolivia: a cross-sectional field study. Parasit Vectors.

[25] Ochoa-Diaz MM, Orozco-Garcia D, Fernandez-Vasquez RS, Eyes-Escalante M (2024). Knowledge, attitudes and practices of Chagas, a neglected tropical disease, in rural communities of the Colombian Caribbean: CHAGCOV study. Acta Parasitol.

[26] Souza ICA, Rodrigues FCS, Araújo AP, Souza JMB, Diotaiuti LG, Ferreira RA (2023). Vigilância à saúde da doença de Chagas em municípios endêmicos de Minas Gerais: percepção e conhecimento de profissionais da vigilância entomológica. Physis (Rio J).

[27] Rodrigues FCS, Souza ICA, Araújo AP, Souza JMB, Diotaiuti LG, Ferreira RA (2020). Agentes comunitários de saúde: percepção sobre os serviços de saúde relacionados à doença de Chagas. Cad Saude Colet.

[28] Ramos WTS, Gonçalves MP, Borsagli FGLM, Rocha EPA, Souza FE, Ramos DTS (2024). Doença de Chagas na Serra Geral, Minas Gerais: perfil e conhecimento de “Agentes de Saúde”. Revista Saúde (Sta. Maria).

[29] Almeida MC, Soares JFS, Santos RS, Ribeiro G, Reis RB, Araújo JMB (2026). Knowledge about Chagas disease among Primary Health Care professionals in a municipality located in northeastern Brazil. PLoS Negl Trop Dis.

[30] Ng TWH, Feldman DC (2013). Does longer job tenure help or hinder job performance?. J Vocat Behav.

[31] Abad-Franch F, Vega MC, Rolón MS, Santos WS, Rojas de Arias A (2011). Community participation in Chagas disease vector surveillance: systematic review. PLoS Negl Trop Dis.

[32] Dias JCP, Dias RB (1985). Participação da comunidade no controle da doença de Chagas. Ann Soc Belg Med Trop.

[33] MS - Ministério da Saúde (2013). Portaria nº 2.761, de 19 de novembro de 2013. Institui a Política Nacional de Educação Popular em Saúde no âmbito do Sistema Único de Saúde (PNEPS-SUS).

[34] Dias LP, Montoya CR, Mendonça VJ, Frontin JMC, Calegari Silva TC (2024). Formação continuada sobre os vetores da doença de Chagas para professores de Teresina, Piauí. Revista Foco.

[35] MS - Ministério da Saúde (2018). Parâmetros ACE municípios. https://antigo.saude.gov.br/noticias/722-svs-noticias/42279-parametrosace-municipios.

[36] M’Batna AJ, Mendes NU, Só KS, M’Batna JJ (2020). Ações educativas em atenção primária à saúde: uma proposta para estratégias de saúde da família. Braz J Dev.

[37] Villela MM, Souza JMB, Melo VP, Dias JCP (2007). Vigilância epidemiológica da doença de Chagas em programa descentralizado: avaliação de conhecimentos e práticas de agentes municipais em região endêmica de Minas Gerais, Brasil. Cad Saude Publica.

[38] MS/CNS - Ministério da Saúde/Conselho Nacional de Saúde (2018). Resolução nº 588, de 12 de julho de 2018. Institui a Política Nacional de Vigilância em Saúde (PNVS). Diário Oficial da União.

[39] Furtado C (1981). O Brasil pós-milagre.

[40] Giambiagi F, Castro LB, Villela AA, Hermann J (2016). Economia brasileira contemporânea: 1945-2015.

[41] Brasil. Constituição (1988) (1988). Constituição da República Federativa do Brasil de 1988.

[42] Rossi AS (2001). Caring and doing for others: social responsibility in the domains of family, work, and community.

[43] Paz MDA, Meireles ACA, Galvão C, Gil-Santana HR, Julião GR (2025). “WhatsBarb” citizen surveillance: survey of insects mistaken for triatomines. Rev Soc Bras Med Trop.

[44] Gurgel-Gonçalves R, Julião GR, Ferreira RA, Souza RCM, Rojas-Cortez M, Vieira TM (2026). New technologies for identification and surveillance of Chagas disease vectors. Rev Soc Bras Med Trop.

