# Supplementary material for: Popular surveillance of Chagas disease and volunteer Collaborators of Triatomine Information Posts operating in Minas Gerais, Brazil, located in municipalities at high risk of triatomine reinfestation
Source: Mem Inst Oswaldo Cruz. 2026 Nov 13;121(Suppl 2):e260107. doi: 10.1590/0074-02760260107 (PMC13765267; doi:10.1590/0074-02760260107)
Supplement: Supplementary file 1 [file 1678-8060-mioc-121-s2-e260107-s1.pdf]

TABLE

Absolute values and percentages of responses to questions related to Chagas disease and its vector

Parameter	Variable	Absolute (N)	Percentage (%)
Knowledge of triatomines	Yes	76	95.00
	No	4	5.00
Feeding preference of triatomines	Plant sap	1	1.25
	Blood of animals and humans	76	95.00
	Only human blood	0	-
	Hemolymph of other insects	0	-
	Others	0	-
	Invalid response	3	3.75
Life cycle stages of triatomines	Egg and adult	8	10.00
	Egg, pupa, nymph and adult	8	10.00
	Egg, nymph and adult	61	76.25
	Other	1	1.25
	No response	2	2.50
Triatomine hiding places*	Behind cabinets, wardrobes, dressers	53	-
	Behind calendars, photos, posters, and papers on walls	32	-
	In cracks and holes in walls	73	-
	On tables, stoves, sofas	6	-
	Between mattresses	49	-
Transmission of the parasite causing Chagas disease by triatomines	Through urine and contaminated faeces	8	10.00
	Through saliva during the bite	13	16.25
	Only through contaminated faeces	57	71.25
	Other	2	2.50

*For this question, respondents could mention more than one answer, making it impossible to calculate percentages. N: number.
