# Supplementary material for: Popular surveillance of Chagas disease and volunteer Collaborators of Triatomine Information Posts operating in Minas Gerais, Brazil, located in municipalities at high risk of triatomine reinfestation
Source: Mem Inst Oswaldo Cruz. 2026 Nov 13;121(Suppl 2):e260107. doi: 10.1590/0074-02760260107 (PMC13765267; doi:10.1590/0074-02760260107)
Supplement: Peer review history

# Reviewer report: FIRST REVIEW ROUND - REVIEWERS’ COMMENTS

## REVIEWER #1

a) Adequacy of the abstract

The abstract is adequate and clearly reflects the objectives, methods, and main findings of the study.

b) Originality and importance of the contribution

The manuscript addresses a relevant topic and presents an original contribution to the field. The introduction clearly presents the research problem and reviews the current state of knowledge. However, the discussion regarding knowledge of triatomines and Chagas disease would benefit from stronger referencing:

Cabrera R, Mayo C, Suárez N, Infante C, Náquira C, García-Zapata MT. Conocimientos, actitudes y prácticas sobre la enfermedad de Chagas en población escolar de una zona endémica del Perú [Knowledge, attitudes, and practices concerning Chagas disease in schoolchildren from an endemic area in Peru]. Cad Saude Publica. 2003 Jan-Feb;19(1):147-54.

Parisi S, Navarro M, Du Plessis JD, Shock JP, Apodaca Michel B, Lucuy Espinoza M, Terán C, Calizaya Tapia NA, Oltmanns K, Baptista Mora A, Saveedra Irala C, Rivera Rojas AA, Rubilar G, Zoller T, Pritsch M. “We have already heard that the treatment doesn’t do anything, so why should we take it?”: A mixed method perspective on Chagas disease knowledge, attitudes, prevention, and treatment behaviour in the Bolivian Chaco. PLoS Negl Trop Dis. 2020 Oct 29;14(10):e0008752.

Salm A, Gertsch J. Cultural perception of triatomine bugs and Chagas disease in Bolivia: a cross-sectional field study. Parasit Vectors. 2019 Jun 10;12(1):291.

Ochoa-Diaz MM, Orozco-Garcia D, Fernandez-Vasquez RS, Eyes-Escalante M. Knowledge, Attitudes and Practices of Chagas a Neglected Tropical Disease in Rural Communities of the Colombian Caribbean, CHAGCOV Study. Acta Parasitol. 2024 Jun;69(2):1148-1156.

Maeda, Maicon Hitoshi ; Knox, Monique Britto ; Gurgel-Gonçalves, Rodrigo . Occurrence of synanthropic triatomines (Hemiptera: Reduviidae) in the Federal District of Brazil. Revista da Sociedade Brasileira de Medicina Tropical (Impresso) , v. 45, p. 71-76, 2012.

It is also unclear whether the workers are aware of currently available surveillance Technologies (described in Gurgel-Goncalves et al. 2026); this aspect should be clarified and, if possible, expanded.

c) Methodology, results, and discussion

The first sentence of the Methods section should be revised, as there is no clear need to justify the exploratory nature of the study. Additionally, the second paragraph consists of only one sentence and should be merged with the previous paragraph, since single-sentence paragraphs are generally not appropriate in scientific writing.

Regarding the Results and Discussion, the participants’ knowledge of Chagas disease and triatomines (Supplementary Table 1) should be more thoroughly explored in the main text. Although the manuscript is somewhat lengthy and could be made more concise, I recommend including a dedicated section or subsection addressing this topic, with a more detailed analysis of Supplementary Table 1. Currently, this information is summarized in a single sentence at the end of the Sociodemographic Profile section, which is insufficient and should be expanded.

d) References

The references are generally appropriate; however, the last reference cited in the manuscript should be updated, as it has already been published.

e) Figures and tables

The figures and tables are adequate and well presented.

## REVIEWER #2

General comments

The manuscript presents an original characterization on the sociodemographic profile of volunteers collaborators from triatomine information posts in Minas Gerais, in areas of high risk for Chagas Disease. The study is an important contribution to improve the monitoring of Chagas disease. The manuscript is very clear, well written and presented. However, before its final acceptance for publication, several corrections must be carried out.

Specific comments

I think the initials for Triatomine Information Post (PIT) should start in the abstract. The one in the title could be crossed out.

The title would be more fluent inverting the terms, please check the suggestion bellow:

“Popular surveillance of Chagas disease and volunteer Collaborators of Triatomine Information Posts operating in Minas Gerais, Brazil, located in municipalities at high risk of triatomine reinfestation”

The authors must decide if the acronym is going to be PIT or TIP, please check the first lines of the abstract

Lines 36-39- In the sentence “…VCs working in TIPs located in rural areas of municipalities in Minas Gerais, Brazil, belonging to areas at high risk of domiciliary reinfestation by triatomines.” Inverting the order of some words would bring more fluency, I think. Please check: “VCs working in TIPs located in rural areas at high risk of domiciliary reinfestation by triatomines in municipalities of Minas Gerais, Brazil.”

I think the sentence “Most VCs reported receiving few insects, due to low community demand for TIPs” My questions, if in the beginning of the abstract the authors mention “at high risk of domiciliary reinfestation by triatomines” why few lines below is mentioned “few insects due to low demand”? This must be well explained and discussed

Introduction

Before starting with the Triatomine Information Post bachground, we recommend presenting a bit of the triatomine diversity in Brazil and principally in the Minas Gerais State. Suggested references:

Costa J, Dale C, Galvão C, Almeida CE, Dujardin JP. Do the new triatomine species pose new challenges or strategies for monitoring Chagas disease? An overview from 1979-2021. Mem Inst Oswaldo Cruz. 2021;116:e210015. pmid:34076075

Gurgel-Gonçalves R, Galvão C, Costa J, Peterson AT. Geographic distribution of chagas disease vectors in Brazil based on ecological niche modeling. J Trop Med. 2012;2012:705326. pmid:22523500 Costa J, Almeida CE, Dotson EM, Lins A, Vinhaes M, Silveira AC, et al. The epidemiologic importance of Triatoma brasiliensis as a chagas disease vector in Brazil: a revision of domiciliary captures during 1993-1999. Mem Inst Oswaldo Cruz. 2003;98(4):443–9. pmid:12937751

Galvão C. 2025. Triatomine Bugs: History, Control, and Citizen Surveillance. Disease Biology, Genetics, and Socioecology 1 (3), 8. https://doi.org/10.53941/dbgs.2025.100008.

Lines 9-10 – Please include the link refered to “Controle da Doença de Chagas, Diretrizes Técnicas”

LIne 24 – Please remove from the text “Triatomine Information Posts” as the initials had already presented before.

Page 3 line 36- Please revise all text to the use of VC. The initials should be explained in the text in the beginning and just once.

M&M

I think the first paragraph of the M&M should be presented in the introduction before the objectives of in the discussion session. The text of the mentioned paragraph is not directly related to the M&M.

Page 4 line 60- Please include a reference on the ” 123 municipalities in Minas Gerais are considered at high risk of domiciliary reinfestation by triatomines in domiciliary units”. Besides that, there is a redundancy. Please check the terms “domiciliary” repeated twice.

Page 5 line 14 “Amaral et al. (2025),” should be referred as a number.

Study area

“of the five health regions located in an area at high risk of domiciliary reinfestation by triatomines in Minas Gerais, comprising 123 municipalities.” This information is repeated from previous paragraph. Please correct

Page 5 Line 69 – PIT or TIP please check the whole text

Results

Text of the results is too long. I think the manuscript could be improved keeping just key information in the results. It is too descriptive.

Discussion

Page15 line 34- The authors show the importance of Health Education, in the study area presented as a high-risk area for Chagas disease. In this sense, it would be important to mention and discuss the paper published based on the teachers’ perception and knowledge on Chagas disease.

FORMAÇÃO CONTINUADA SOBRE OS VETORES DA DOENÇA DE CHAGAS PARA PROFESSORES DE TERESINA, PIAUÍ

LP Dias, CR Montoya, VJ Mendonça, JMC de Frontin… - REVISTA FOCO, 2024

Legends

“S1 Fig. Spatial distribution of TIPs based on the type of installation site.” This legend is written twice. Please check the correct one and cross out the other.

There is no appropriate legend for the map. I think the map should be presented as Fig 1 not as a supplementary material

Legends must be improved. All legends must be self-explanatory. Please revise all of them providing detailed information in all of them, complementing their text according to the appropriate rules and additional information.

## AUTHORS’ RESPONSE TO THE REVIEWERS

Dear reviewers,

We appreciate your suggestions and accept most of them. Below are our point-by-point responses. We would like to highlight that all additions made to the manuscript text have been indicated in colored font, and all deletions have been marked using the Word track changes feature.

## Reviewer: 1

a) Adequacy of the abstract.

The abstract is adequate and clearly reflects the objectives, methods, and main findings of the study.

Authors: We appreciate your positive feedback.

b) Originality and importance of the contribution

The manuscript addresses a relevant topic and presents an original contribution to the field. The introduction clearly presents the research problem and reviews the current state of knowledge. However, the discussion regarding knowledge of triatomines and Chagas disease would benefit from stronger referencing:

Cabrera R, Mayo C, Suárez N, Infante C, Náquira C, García-Zapata MT. Conocimientos, actitudes y prácticas sobre la enfermedad de Chagas en población escolar de una zona endémica del Perú [Knowledge, attitudes, and practices concerning Chagas disease in schoolchildren from an endemic area in Peru]. Cad Saude Publica. 2003 Jan-Feb;19(1):147-54.

Parisi S, Navarro M, Du Plessis JD, Shock JP, Apodaca Michel B, Lucuy Espinoza M, Terán C, Calizaya Tapia NA, Oltmanns K, Baptista Mora A, Saveedra Irala C, Rivera Rojas AA, Rubilar G, Zoller T, Pritsch M. “We have already heard that the treatment doesn’t do anything, so why should we take it?”: A mixed method perspective on Chagas disease knowledge, attitudes, prevention, and treatment behaviour in the Bolivian Chaco. PLoS Negl Trop Dis. 2020 Oct 29;14(10):e0008752.

Salm A, Gertsch J. Cultural perception of triatomine bugs and Chagas disease in Bolivia: a cross-sectional field study. Parasit Vectors. 2019 Jun 10;12(1):291.

Ochoa-Diaz MM, Orozco-Garcia D, Fernandez-Vasquez RS, Eyes-Escalante M. Knowledge, Attitudes and Practices of Chagas a Neglected Tropical Disease in Rural Communities of the Colombian Caribbean, CHAGCOV Study. Acta Parasitol. 2024 Jun;69(2):1148-1156.

Maeda, Maicon Hitoshi ; Knox, Monique Britto ; Gurgel-Gonçalves, Rodrigo . Occurrence of synanthropic triatomines (Hemiptera: Reduviidae) in the Federal District of Brazil. Revista da Sociedade Brasileira de Medicina Tropical (Impresso) , v. 45, p. 71-76, 2012.

It is also unclear whether the workers are aware of currently available surveillance Technologies (described in Gurgel-Goncalves et al. 2026); this aspect should be clarified and, if possible, expanded.

Authors: We appreciate your comments. We added the topic “Aspects related to Chagas disease and its vectors” to the Discussion section and incorporated most of the references suggested, as recommended.

Regarding your last observation, we did not assess whether the community volunteers were familiar with the surveillance technologies mentioned in the cited study (Gurgel-Goncalves et al. 2026); except for the TIPs. Therefore, we regret that we are unable to include any information on this matter in the manuscript.

c) Methodology, results, and discussion

The first sentence of the Methods section should be revised, as there is no clear need to justify the exploratory nature of the study. Additionally, the second paragraph consists of only one sentence and should be merged with the previous paragraph, since single-sentence paragraphs are generally not appropriate in scientific writing.

Authors: The first sentence of the Methods section was removed, and the second paragraph was merged with the following one, as suggested.

Regarding the Results and Discussion, the participants’ knowledge of Chagas disease and triatomines (Supplementary Table 1) should be more thoroughly explored in the main text. Although the manuscript is somewhat lengthy and could be made more concise, I recommend including a dedicated section or subsection addressing this topic, with a more detailed analysis of Supplementary Table 1. Currently, this information is summarized in a single sentence at the end of the Sociodemographic Profile section, which is insufficient and should be expanded.

Authors: We thank the reviewer for the comments and expanded this topic as suggested.

d) References

The references are generally appropriate; however, the last reference cited in the manuscript should be updated, as it has already been published.

Authors: We thank the reviewer for the observation and updated the reference.

e) Figures and tables

The figures and tables are adequate and well presented.

Authors: We thank the reviewer for the comments

## Reviewer: 2

General comments

The manuscript presents an original characterization on the sociodemographic profile of volunteers collaborators from triatomine information posts in Minas Gerais, in areas of high risk for Chagas Disease. The study is an important contribution to improve the monitoring of Chagas disease. The manuscript is very clear, well written and presented. However, before its final acceptance for publication, several corrections must be carried out.

Authors: We appreciate your positive feedback.

Specific comments

I think the initials for Triatomine Information Post (PIT) should start in the abstract. The one in the title could be crossed out.

Authors: We appreciate and readily accept your suggestion.

The title would be more fluent inverting the terms, please check the suggestion bellow:

“Popular surveillance of Chagas disease and volunteer Collaborators of Triatomine Information Posts operating in Minas Gerais, Brazil, located in municipalities at high risk of triatomine reinfestation”

Authors: We appreciated and accepted your title suggestion.

The authors must decide if the acronym is going to be PIT or TIP, please check the first lines of the abstract

Authors: We appreciate your careful observation, and we standardized the abbreviations throughout the text to TIP.

Lines 36-39- In the sentence “…VCs working in TIPs located in rural areas of municipalities in Minas Gerais, Brazil, belonging to areas at high risk of domiciliary reinfestation by triatomines.” Inverting the order of some words would bring more fluency, I think. Please check: “VCs working in TIPs located in rural areas at high risk of domiciliary reinfestation by triatomines in municipalities of Minas Gerais, Brazil.”

Authors: The sentence was revised as suggested.

I think the sentence “Most VCs reported receiving few insects, due to low community demand for TIPs” My questions, if in the beginning of the abstract the authors mention “at high risk of domiciliary reinfestation by triatomines” why few lines below is mentioned “few insects due to low demand”? This must be well explained and discussed

Authors: Dear reviewer, your question is very interesting and pertinent. We believe that, after reading the manuscript, you noticed that although the municipalities are located in an area with a high risk of household reinfestation by triatomines, there is low community demand for the TIPs. In fact, this contradiction is one of the most relevant aspects discussed in the study. In this regard, we kept this information in the abstract.

Introduction

Before starting with the Triatomine Information Post bachground, we recommend presenting a bit of the triatomine diversity in Brazil and principally in the Minas Gerais State. Suggested references:

Costa J, Dale C, Galvão C, Almeida CE, Dujardin JP. Do the new triatomine species pose new challenges or strategies for monitoring Chagas disease? An overview from 1979-2021. Mem Inst Oswaldo Cruz. 2021;116:e210015. pmid:34076075

Gurgel-Gonçalves R, Galvão C, Costa J, Peterson AT. Geographic distribution of chagas disease vectors in Brazil based on ecological niche modeling. J Trop Med. 2012;2012:705326. pmid:22523500

Costa J, Almeida CE, Dotson EM, Lins A, Vinhaes M, Silveira AC, et al. The epidemiologic importance of Triatoma brasiliensis as a chagas disease vector in Brazil: a revision of domiciliary captures during 1993-1999. Mem Inst Oswaldo Cruz. 2003;98(4):443–9. pmid:12937751

Galvão C. 2025. Triatomine Bugs: History, Control, and Citizen Surveillance. Disease Biology, Genetics, and Socioecology 1 (3), 8. https://doi.org/10.53941/dbgs.2025.100008.

Authors: Dear reviewer, we appreciate your suggestion and carefully reflected on it. However, we decided not to incorporate it, considering that the introduction already begins by addressing entomological surveillance, and the addition of information regarding triatomine diversity would disrupt the homogeneity and flow of the section. Furthermore, we do not consider this information to be essential to the manuscript.

Lines 9-10 – Please include the link refered to “Controle da Doença de Chagas, Diretrizes Técnicas”

Authors: Dear reviewer, thank you for your observation. However, the document “Controle da Doença de Chagas: Diretrizes Técnicas” (FUNASA/MS, 1994) is an old publication, and the official PDF from the Ministry of Health is currently not available on the existing government portals. Therefore, it is not possible to provide a link as requested.

LIne 24 – Please remove from the text “Triatomine Information Posts” as the initials had already presented before.

Authors: We accepted your suggestion.

Page 3 line 36- Please revise all text to the use of VC. The initials should be explained in the text in the beginning and just once.

Authors: The entire text was revised and the appropriate adjustments were made.

M&M

I think the first paragraph of the M&M should be presented in the introduction before the objectives of in the discussion session. The text of the mentioned paragraph is not directly related to the M&M.

Authors: Dear reviewer, we appreciate your observation and, in accordance with the first reviewer’s suggestion, we decided to remove this paragraph from the manuscript.

Page 4 line 60- Please include a reference on the ” 123 municipalities in Minas Gerais are considered at high risk of domiciliary reinfestation by triatomines in domiciliary units”. Besides that, there is a redundancy. Please check the terms “domiciliary” repeated twice.

Authors: We appreciate your observation. However, unfortunately, there is no official publication presenting the risk stratification for the state of Minas Gerais. There are only internal documents from the State Health Department of Minas Gerais and stratification maps made available by the department. Therefore, we apologize, but we will not be able to comply with your request.

Authors: We addressed the reviewer’s comment and removed the word “domiciliary.”

Page 5 line 14 “Amaral et al. (2025),” should be referred as a number.

Authors: We appreciate your suggestion, but we believe that modifying the structure of this sentence would not be appropriate. Therefore, we retained Amaral, 2025, which is reference number 8.

Study area“of the five health regions located in an area at high risk of domiciliary reinfestation by triatomines in Minas Gerais, comprising 123 municipalities.” This information is repeated from previous paragraph. Please correct

Authors: We appreciate your comment, and the text was removed.

Page 5 Line 69 – PIT or TIP please check the whole text

Authors: The entire text was revised according to your guidance.

Results

Text of the results is too long. I think the manuscript could be improved keeping just key information in the results. It is too descriptive.

Authors: We appreciate your consideration and carefully reflected on it. However, given the nature of the study, we decided to keep the results highly descriptive and detailed, as we believe that reducing the content could compromise the understanding of the data.

Discussion

Page15 line 34- The authors show the importance of Health Education, in the study area presented as a high-risk area for Chagas disease. In this sense, it would be important to mention and discuss the paper published based on the teachers’ perception and knowledge on Chagas disease.

FORMAÇÃO CONTINUADA SOBRE OS VETORES DA DOENÇA DE CHAGAS PARA PROFESSORES DE TERESINA, PIAUÍ

Authors: We appreciate the excellent suggestion and have added the article to the discussion section.

Legends

“S1 Fig. Spatial distribution of TIPs based on the type of installation site.” This legend is written twice. Please check the correct one and cross out the other.

Authors: We appreciate your observation, and we removed one of the legends.

There is no appropriate legend for the map. I think the map should be presented as Fig 1 not as a supplementary material

Legends must be improved. All legends must be self-explanatory. Please revise all of them providing detailed information in all of them, complementing their text according to the appropriate rules and additional information.

Authors: We appreciate your careful review, and we revised the map legend and improved the other figure legends as well.

# Reviewer report: REVIEWERS’ COMMENTS

## REVIEWER #1

The manuscript was improved and should be accepted.

## REVIEWER #2

The manuscript was greatly improved.

However, figure 1 was not found in the text.

In the reviewed manuscript, in the legends, it seems there is a mistake in the corrected fig 2. Please check the legends. Finaly, I think in the introduction, brief words on CD and the vectors in the studied area would bring a broad context for the manuscript. The authors did not agree. Thefore, I leave the decision with the editors.
